# Life-threatening acute acalculous cholecystitis in a patient with renal cell carcinoma treated by sunitinib: a case report

**DOI:** 10.1186/1752-1947-6-69

**Published:** 2012-02-20

**Authors:** Kazuhiko Nakano, Kazumi Suzuki, Tatsuo Morita

**Affiliations:** 1 Department of Urology, Jichi Medical University, Yakushiji 3311-1, Shimotsuke-city, Tochigi 3290498, Japan

## Abstract

**Introduction:**

Sunitinib, an oral multitargeted tyrosine kinase inhibitor, is widely used in the treatment of renal cell carcinoma and gastrointestinal stromal tumor and has had a variety of adverse events. However, sunitinib-related acute cholecystitis has been reported in only two patients with gastrointestinal stromal tumor and renal cell carcinoma (clear cell subtype).

**Case presentation:**

A 75-year-old Japanese woman with a right sided abdominal swelling was referred to our hospital. Computed tomography (CT) showed a hypervascular bulky tumor in her right kidney, suggesting right renal cell carcinoma in clinical T4N0M0. Although sunitinib therapy was started as neoadjuvant chemotherapy, during the fourth week of the first cycle, she developed acute acalculous cholecystitis and disseminated intravascular coagulation associated with sunitinib. Sunitinib therapy was discontinued immediately and she recovered after subsequent treatment with antibiotics and gabexate mesilate followed by percutaneous cholecystostomy. Cholecystectomy and right radical nephrectomy were performed and pathological examination showed that her renal tumor was a chromophobe renal cell carcinoma (pT2) with necrosis. Inflammation and ischemia were observed in the gallbladder wall, which was compatible with acute acalculous cholecystitis. There has been no evidence of disease recurrence for more than six months.

**Conclusion:**

We described the third case of sunitinib-related acute cholecystitis in a patient with chromophobe renal cell carcinoma. Attention is required to sunitinib-related acute cholecystitis which, while uncommon, could be life-threatening.

## Introduction

Sunitinib, an oral multitargeted tyrosine kinase inhibitor, is widely used in the treatment of metastatic renal cell carcinoma (RCC) and gastrointestinal stromal tumor (GIST) and has been administered in the perioperative period [1]. Although sunitinib has had a variety of adverse events, sunitinib-related acute cholecystitis has been reported in only two patients with GIST and RCC (clear cell subtype). We report a third case of sunitinib-related acute cholecystitis in a patient with chromophobe RCC who developed a serious condition.

## Case presentation

**Figure 1:**
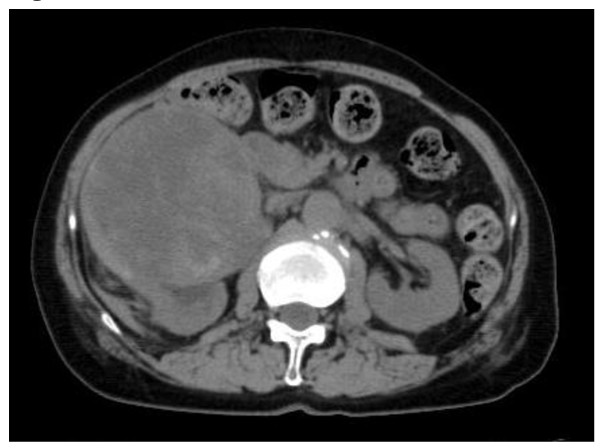
**Abdominal CT showed right renal mass, suggestive of RCC at the first visit**.

**Figure 2:**
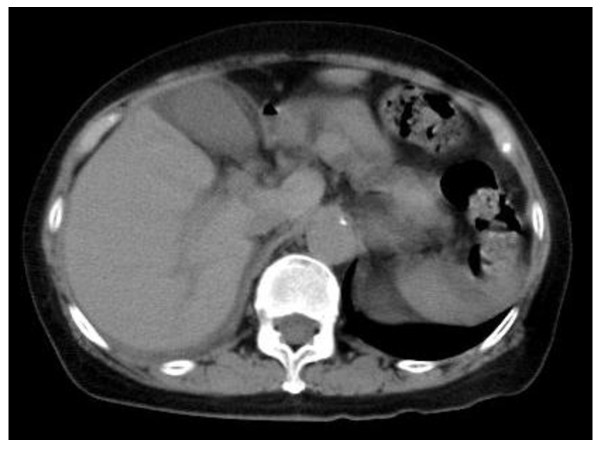
**Abdominal CT showed a normal gallbladder at the first visit**.

**Figure 3:**
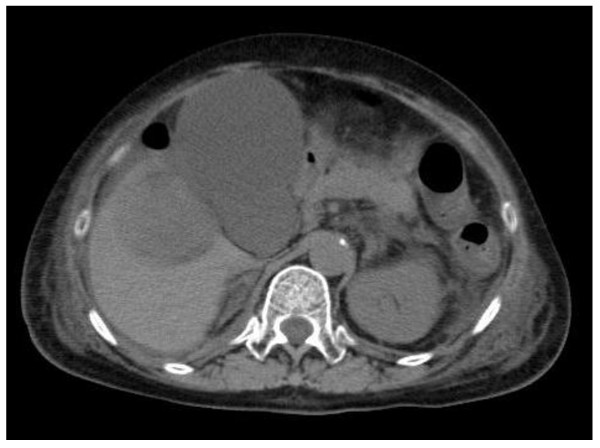
**Abdominal CT showed acalculous cholecystitis during the fourth week of the first cycle of sunitinib therapy**.

A 75-year-old Japanese woman with a right sided abdominal swelling was referred to our hospital. She had no history of medication or smoking and was a social drinker. Computed tomography (CT) showed a hypervascular bulky tumor in her right kidney with suspected liver invasion without distant metastasis (Figure 1), suggesting right RCC in clinical T4N0M0. For the purpose of downstaging of the tumor, sunitinib therapy (50 mg per day, four weeks on and two weeks off) was started in the neoadjuvant setting. During the fourth week of the first cycle, she experienced right upper quadrant pain with a positive Murphy's sign and abdominal fullness without fever. Laboratory tests revealed elevated levels of C-reactive protein, lactate dehydrogenase, and liver transaminases although total bilirubin, alkaline phosphatase, and amylase were at normal levels. She also had laboratory features of disseminated intravascular coagulation (DIC) including thrombocytopenia and disordered coagulation. Despite a normal gallbladder at the first visit (Figure 2), abdominal computed tomography (CT) revealed a tense and dilated gallbladder and thickening of the gallbladder wall without gallbladder stones or emphysematous change (Figure 3). Based on the diagnosis of acute acalculous cholecystitis associated with sunitinib, sunitinib therapy was discontinued immediately. She recovered in an intensive care unit after subsequent treatment with antibiotics and gabexate mesilate (FOY^®^) followed by percutaneous cholecystostomy.

**Figure 4:**
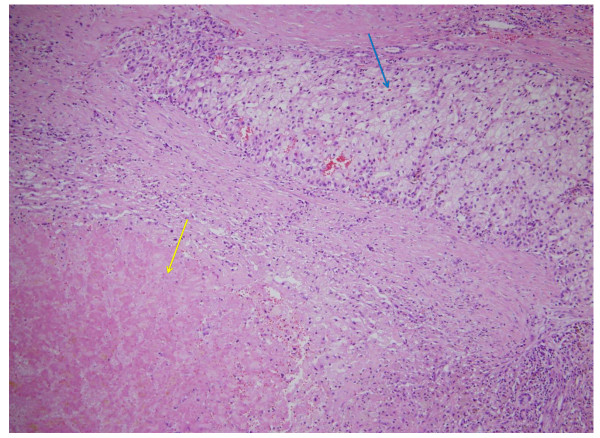
**Chromophobe renal cell carcinoma (RCC) (blue arrow) with necrosis (yellow arrow)**.

**Figure 5:**
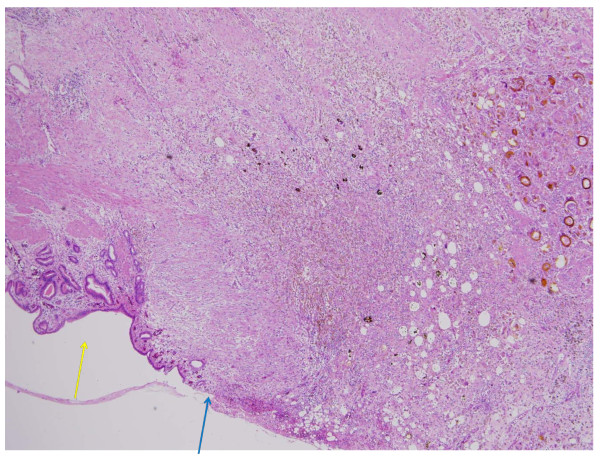
**Gallbladder epithelium showed inflammation with necrosis (blue arrow) and normal epithelium in part (yellow arrow)**.

After three months, a follow-up contrast-enhanced computed tomography (CT) revealed a marked shrinkage of the gallbladder and a 21% reduction in the size of the renal tumor with decreased enhancement of its center. Cholecystectomy and right radical open nephrectomy were performed. Adhesions thought to be due to cholecystitis made the operation difficult although common bile duct stenosis or retraction by the tumor that could lead to cholecystitis was not observed. Pathological examination showed that her renal tumor was chromophobe RCC (pT2) with necrosis occupying more than half of the tumor (Figure 4). Inflammation and ischemia were observed in the gallbladder wall which was compatible with acute acalculous cholecystitis (Figure 5). Computed tomography (CT) has revealed no evidence of disease recurrence for more than six months since the radical nephrectomy.

## Discussion

We present the third case of sunitinib-related acute cholecystitis which developed in a patient with a chromophobe RCC. In the literature, sunitinib-related acute cholecystitis has been reported in only two other patients, one with GIST [2] and the other with RCC (clear cell subtype) [3]. We evaluated whether or not acute cholecystitis in our patient was caused by sunitinib with the use of the Naranjo scale [4] which assesses the probability of a drug-related adverse event [2,3]. Its scale score for our patient was five (Table 1), indicating a probable association of acute cholecystitis with sunitinib. Sunitinib-related acute cholecystitis was also supported by the following findings: the symptoms improved with discontinuation of sunitinib; there were no risk factors including gallbladder stones, common bile duct stenosis and other hepatobiliary diseases that could cause acute cholecystitis and deranged liver function.

**Table 1:** Adverse drug reaction probability scale (Naranjo Scale) in the present case

	Yes	No	Don't know	Score
1. Are there previous conclusive reports on this reaction?		✓		0
2. Did the adverse event appear after the suspected drug was given?	✓			2
3. Did the adverse reaction improve when the drug was discontinued or a specific antagonist was given?			✓	0
4. Did the adverse reaction appear when the drug was readministered?			✓	0
5. Are there alternative causes that could have caused the reaction?		✓		2
6. Did the reaction reappear when a placebo was given?			✓	0
7. Was the drug detected in any body fluid in toxic concentrations?			✓	0
8. Was the reaction more severe when the dose was increased, or less severe when the dose was decreased?			✓	0
9. Did the patient have a similar reaction to the same or similar drugs in any previous exposure?			✓	0
10. Was the adverse event confirmed by any objective evidence?	✓			1
Scoring				5

A common clinical feature of sunitinib-related acute cholecystitis in three cases including our patient and the previous two cases [2,3] was acalculous cholecystitis while gallbladder stones have been found in 90% of patients with acute cholecystitis [5]. On the other hand, sunitinib causes vascular adverse events by vascular endothelial dysfunction [6,7] resulting in myocardial ischemia [8] and proteinuria due to renal thrombotic microangiopathy [9]. Likewise, sunitinib-related acute cholecystitis might be caused by the antivascular effect of sunitinib rather than gallbladder stones although the detailed mechanism underlying this adverse event is unclear. As sunitinib is widely used in the treatment of RCC and GIST, we need to pay attention to sunitinib-related acute cholecystitis which, while uncommon, could be life-threatening.

## Conclusion

We described the third case of sunitinib-related acute cholecystitis in a patient with chromophobe RCC. Attention needs to be given to sunitinib-related acute cholecystitis which could be life-threatening albeit uncommon.

## Consent

Written informed consent was obtained from the patient for publication of this case report and any accompanying images. A copy of the written consent is available for review by the Editor-in-Chief of this journal.

## Competing interests

The authors declare that they have no competing interests.

## Authors' contributions

KN prepared the manuscript. KS and TM interpreted the patient data and revised the manuscript. All authors reviewed and approved the final version of the manuscript.
